# Prof. Meynert’s Method of Examining the Brain, Etc.

**Published:** 1873-11

**Authors:** James J. Putnam


*Prof. Meynert's Method of Examining the Brain, etc.* By James J. Putnam, M.D.

In an interesting paper by Dr. Burt G. Wilder, entitled Cynophrenology, which appeared in the Journal of January 23, ult., allusion is made to the insufficiency of our present methods of gauging the relative excellence of the different mammal brains.

It is remarked with truth that neither the absolute size of the brain, nor its relative size as compared with the whole animal, nor the number or complexity of its fissures, satisfactorily indicates its quality. The first test would place the brain of the elephant and the whale too high in the scale, the second, too low.

There is, however, one test, first suggested, as I believe, by Prof. Meynert, of Vienna, which seems to be a satisfactory and practical one. That is, stated briefly, the relation in point of size or weight between the corp. quadrigem. and the cerebral lobes; or, in other words, the relation in point of development between the so-called "posterior division" * of the caudex cerebri (Hirnstamm) with its ganglia of origin, and the "anterior division" with its ganglia, *i. e.*, between the tegmentum cruris cerebri with the thalamus opticus and the corp. quadrigem., and the pes (or basis) cruris cerebri with the corpus striatum and nucleus lenticularis.

This latter tract (the pes cruris) is concerned according to Prof. Meynert, almost exclusively in the transmission of voluntary impulses to the muscles, and stands, therefore, in a fixed relation to the cerebral lobes, in which the voluntary impulses are originated, throughout the entire mammal series. The development of these two structures, viz., pes cruris and cerebral lobes, culminates in the brain of the adult man, whereas the tegmentum cruris with the corp. quad., etc., find their greatest development in the brains of the lower mammals, as the following table will indicate:

| | Man. Per cent. | Ape. Per cent. | Deer. Per cent. |
|---|---|---|---|
| Height of basis cruris cer. compared with that of tegmentum | 1.1 | 1.3 | 1.5 |
| Weight of cerebral lobes compared with that of entire brain | 78 | 70.8 | 62 |
| Weight of corpus striatum, with the nucleus lenticularis, compared with that of the entire caudex cerebri | 50 | 40 | 33.3 |
| Weight of thalamus opticus compared with that of the entire caudex cerebri | 19 | 22.9 | 30 |

* Compare: The Anatomy of the Mammal Brain, by Theodor Meynert, Stricker's Manual of Histology, American edition; also, an analysis of the same in Brown-Sequard's Archives of Scientific and Practical Medicine for February, 1873; also, Ueber die Bedeutung der Zweifachen Ruckenmarksursprunges vom Grosshirn. Sitzungsberichte der Koniglichen Academie der Wissenschaften. Bd. lxi., H. iii.

A table is annexed to the paper "Ueber die Bedeutung des Zweifachen Ursprunges des Ruckenmarkes, etc.," showing that, among the large number of mammals examined with reference to that point, in none did the development of the pes cruris equal that to which the corresponding part of the human brain attains.

This seems a not unsuitable place for recording Prof. Meynert's manner of examining the brain in the autopsy room, which is of value because it permits, more than any other, of the exact localization of defined lesions, without at the same time so mutilating the parts as to make them unfit for subsequent microscopic or other examination. The method aims, in the first instance, at the enucleation of the cerebral ganglia from their bed in the white substance of the lobes, so that all the surfaces of these parts may be freely examined.

Each cerebral hemisphere, according to Prof. Meynert, presents at both surfaces the form of an arch, of which one branch is represented by the frontal extremity, the other by the temporal extremity of the hemisphere.

The lumen of the arch of the inner surface is the lateral ventricle, of that of the outer surface the fissure of Sylvius. At the bottom of the fissure of Sylvius lies the island of Reil; directly beneath, and scarcely separated from this, is the surface of the nucleus lenticularis,* a cone-shaped ganglion which lies crosswise in the axis of the arch. Surrounding this, in a curve concentric with that of the main arch, is the corpus striatum (nucleus caudatus), whose large anterior extremity (caput) reaches forward and downward, buried in the frontal lobe, to the anterior perforated space, where it is nearly met by the dwindling posterior extremity (cauda) which curves backward and downward along the lateral ventricle, in which it is everywhere visible, nearly to the extremity of the temporal lobe.

The proposed dissection throws these two false arches into one true arch by cutting out this ganglionic mass which fills its lumen.

The knife has for its guide, in the fissure of Sylvius, the limits of the island of Reil; in the lateral ventricle, the border of the gray surface of the corpus striatum which runs along its inner angle. The principal details of the operation are as follows: †

The brain, removed in the usual way, is laid down upon its convexity, and, the membranes having been torn away as far as is requisite, the temporal lobe on both sides is dissected upwards by a series of cuts opening into the lateral ventricle on one hand, and the fissure of Sylvius on the other, and made in accordance with the rule given above.

* Nucleus extraventricularis corp. striat.

† Given from memory; I do not know that they are to be found in print.

The next step is to raise the medulla oblongata and cerebellum, and tear away the membranes beneath them which cover the great transverse fissure of the brain. Then, by a second series of cuts made from behind forwards, the mass of ganglia, principally at that part the thalamus opticus, is separated from its connections with the occipital and parietal, and the posterior half of the frontal lobes.

It only remains to enucleate the caput corporis striati from its bed in the white substance of the frontal lobe. This is done by laying the knife transversely on its flat with its edge just in front of the anterior perforated space, and cutting forwards and downwards. If the cut has not been carried forward far enough to surround the bulging head of the ganglion completely, the gray color of its cut surface will reveal the fact. The entire operation, apparently so complicated, is after some practice performed in a very few moments and with great precision. If it be desirable to weigh the different lobes and ganglia separately, as Prof. Meynert is in the habit of doing, the dissection may be continued as follows; otherwise the examination may be completed by making a number of cuts into the various parts perpendicularly to their surface:

A longitudinal cut through the corpus callosum separates the hemispheres from one another.

To separate the frontal lobe from the parietal, the fissure of Rolando, inasmuch as it is a constant fissure common also to most of the apes, is chosen as a guide. The operation is best performed with a single cut of a large pair of scissors.

The parietal lobe is separated from the occipital by cutting through with the scissors from the occipito-parietal fissure into the posterior branch of the fissure of Sylvius.

In separating the thalamus opticus from the corpus striatum, one blade of the scissors is placed to the inner side of the stria cornea, the other to the outer side of the tractus opticus. The cut passes though the capsula interna with little or no injury to either ganglion. The limits of the corp. quadrigem., cerebellum and pons Varolii are sufficiently evident. The medulla oblongata ends with the completion of the decussation of the pyramids.

Prof. Meynert has divided in this way, and weighed in parts, upwards of 1,000 brains, with interesting results, not yet fully made public. Among them, the disproportionate atrophy of the frontal lobes in cases of progressive paralysis of the insane, may be mentioned as having been constantly found. In one or more cases of unilateral chorea, the corpus striatum of the opposite side was found to have lost weight distinctly.

The accuracy of the results obtained in this manner is the more to be relied on, in consideration of the fact that if a loss or gain in weight affecting any given part is only apparent, it will be found compensated for by a corresponding gain or loss in the complementary part, and the observation thereby controlled. The membranes are left adherent to the lobes to which they belong.

The thickness of the cortex cerebri may be estimated by a cut, made always at the same point, and in the same direction.*—*Boston Med. and Surg. Journal.*

* For this purpose a small, graduated glass tube, with sharpened edges, which has recently been described, would be of service. A piece of the cortex is simply punched out, and examined through the glass. (v. West-Riding Hospital Reports for 1873.)

